# Current News and Opinion

**Published:** 1884-07

**Authors:** 


### Current News and Opinion.

### THE DINNER TO DR. MILLER.

It is a good and ancient custom for a body of men, when they wish to show their appreciation of the qualities of another, or to acknowledge their indebtedness to him for light upon dark questions, to give a dinner in his honor. When they have very particular reasons for delighting to honor a colleague they are extremely fortunate, and much more successful in their object if the management of the affair can be in as good hands as was the dinner in the "Diamond Alcove" of the Union League Club House, New York, on Saturday evening, June 21, in honor of Dr. Willoughby D. Miller, of Berlin.

In the five short years since Dr. Miller's graduation he has made for himself a name second to none among original investigators with the microscope, and the profession owes much to his careful, patient study, and the trained eyes which know what they see. The presence of living organisms in dental caries has been known for some years, but that they were such important factors in producing decay has only recently been discovered, and it has been left for Dr. Miller to give a careful and satisfactory explanation of their destroying methods.

All the requisites for a perfect evening were present on Saturday; a distinguished guest, a dignified and Apollo-like toast-master, a faultless *menu*, and an appreciative head for each of the sixteen pairs of feet which were thrust under that noble mahogany. Anecdotes and pleasing jests were heard, some recitations by one who, with equal talent for each, simply prefers dentistry to elocution, speeches of welcome, congratulation, and acknowledgment—off hand they were, but sincere and from the heart—all present being well pleased that one who is doing so much for dental science as Dr. Miller should be so loyally true to his country and countrymen as to desire a position and name in America above all else, and the first publication here of the results of his investigations.

A happy occasion, and one never to be forgotten. S. E. D.

### SUPREME COURT OF ILLINOIS.

OPINION FILED MAY 19, 1884.

THE PEOPLE OF THE STATE OF ILLINOIS *ex rel.* ISAAC A. SHEPARD *v.* THE ILLINOIS STATE BOARD OF DENTAL EXAMINERS.

*Petition for Mandamus.*

### POWER OF BOARD—LICENSE TO PRACTICE DENTISTRY.

1. *Held*, That the board has power to determine whether the applicant for license has graduated from a "reputable dental college"; that in making this determination it acts in a judicial capacity, and from its decision in this respect there is no appeal.

2. MANDAMUS.—That in certain cases, the court could by mandamus compel the board to act, but it could not compel it to act in a particular manner. —*Ed. Chicago Legal News.*

Opinion by SCHOLFIELD, J. It is provided by the first section of an act approved May 30, 1881, entitled "An act to insure the better education of practitioners of dental surgery, and to regulate the practice of dentistry in the State of Illinois," "that it shall be unlawful for any person who is not at the time of the passage of this act engaged in the practice of dentistry in this State, to commence such practice unless such person shall have received a diploma from the faculty of some reputable dental college, duly authorized by the laws of this State, or of some other of the United States, or by the laws of some foreign country, in which college or colleges there was at the time of the issue of such diploma, annually delivered a full course of lectures and instruction in dental surgery * * *." And in the sixth section of the same act, after providing for examination, before the board of dental examiners, of all applicants for license to practice dentistry, is the following provision: "But said board shall, at all times, issue a license to any regular graduate of any reputable dental college without examination upon the payment of such graduate, to the said board, of a fee of one dollar * * *."

Other provisions of the act prohibit any person to practice dentistry without a license from the board, except such as are properly enrolled as having been practitioners at the time of the passage of the act. The contention of the relator is that the board of dental examiners have no power to decide what is or what is not a "reputable dental college;" that the law has, itself, defined what is a "reputable dental college," in providing that it shall be "duly authorized by the laws of this State, or some other of the United States, or by the laws of some foreign country, in which college * * * there was at the time of the issue of such diploma, annually delivered a full course of lectures and instruction in dental surgery."

We are unable to appreciate the force of this position. The word "reputable" would seem to be used here to express the meaning ordinarily attached to it. If it had been intended that a diploma from any dental college, or a diploma from any dental college "duly authorized by the laws of this State, or some other of the United States, or by the laws of some foreign country, in which college * * there was at the time of the issue of such diploma, annually delivered a full course of lectures and instructions in dental surgery," we must presume the language would have so said. By using the word "reputable" we must presume the General Assembly meant "reputable." And since it is not used as being the equivalent and convertible for the other requirements in regard to the college, but as in addition thereto, we must presume it was intended to be so construed. As a part of the current history of the times and as an aid in arriving at the legislative intention, we know there were colleges of different kinds, authorized by the laws of States in which they were located, in which there were pretended to be annually delivered full courses of lectures and instructions upon the arts and sciences professed to be taught, that were not "reputable," because they graduated for money frequently without any reference to scholarship. A diploma from such an institution afforded no evidence of scholarship or attainments in its holder. It was a fraud, and deserved no respect from anybody. And it was as against such diplomas the law was intended to protect the public, and therefore required that the colleges be "reputable." Whether a college be reputable or not is not a legal question, but a question of fact. So, also, are the requirements in regard to the actual delivery of full courses of lectures and instructions. These questions of fact are, by the act, submitted to the decision of the board—not in so many words, but by the plainest and most necessary implication. Their action is to be predicated upon the existence of the requisite facts, and no other tribunal is authorized to investigate them. And of necessity, therefore, they must do so. The act of ascertaining and determining what are the facts, is in its nature judicial. It involves investigation, judgment and discretion. The office of the writ of mandamus is, in general, to compel the performance of mere ministerial acts prescribed by law. It lies, however, also to subordinate judicial tribunals to compel them to act where it is their duty to act, but never to require them to decide in a particular manner. It is not like a writ of error or appeal, a remedy for erroneous decisions: Judges of Oneida Common Pleas *v.* People, 18 Wendell, 92. And as is said by the court in People ex. rel. *v.* Com. Council of Troy, 78 N. Y., 33, this principle applies to every case where the duty, performance of which is sought to be compelled, is in its nature judicial, or involves the exercise of judicial power or discretion, irrespective of the general character of the officer or body to which the writ is addressed. A subordinate body can be directed to act, but not how to act, in a matter as to which it has the right to exercise its judgment. The character of the duty, and not that of the body or officers, determines how far performance of the duty may be enforced by mandamus.

Where a subordinate body is vested with power to determine a question of fact, the duty is judicial, and though it can be compelled by mandamus to determine the fact, it can not be directed to decide it in a particular way, however clearly it be made to appear what the decision ought to be.

See also Kelly et al. *v.* City of Chicago, 62 Ill., 279.

Illustrations of the principle will be found in People *v.* Common Council of Troy *supra;* Freeman *v.* Selectmen, etc., 34 Conn., 406; Hoole *v.* Kinkead, 17 Nevada, 217; Bailey *v.* Ewart, 52 Iowa, 111; Berryman *v.* Perkins, 55 Cal., 483; People *v.* Contracting Board, 27 N. Y., 378, and other cases cited in argument by the Attorney-General.

The demurrer here does not admit that the board of dental examiners found that the college at which the relator was graduated was reputable, although it does admit that to be the fact. But since the board can not be compelled to decide the question that way, although the evidence might clearly sustain it in doing so, there is no ground for mandamus.

The demurrer must be sustained and the petition dismissed.

Petition dismissed.

---

### MEETING OF DELEGATES.

Delegates to the National Dental Association, and others who may act in a representative capacity, will meet in Washington City July 22, at the Ebbitt House, where reduced rates have been made. Reduced railroad rates may be obtained by unity of action on the part of those who will attend from States or sections. Everything indicates a large meeting for organization. Enough States have already elected delegates to insure a good attendance and a profitable meeting.

The acceptability of the place has been shown by the large number of favorable responses from every section of the United States.

The profession of America will unite for one purpose and for one common cause, to-wit: To build up the honor and name of our calling. An organization will be put forth in which self will be lost to the good of the order. Unity and action will be brought about; brotherly love of every section will be united. An association will be formed that will fill the heart of every American dentist with pride and stimulate to higher aims and nobler purposes.

B. H. Catching, Correspondent.

Atlanta, Ga., June 14, 1884.

A consummation devoutly to be wished. But can Dr. Catching give any guarantees concerning the dawn of this professional millennium? We sincerely hope this body will meet and sit. Who knows? It may hatch out a new Cosmic egg. We have but four National Dental Associations now. Surely there must be abundant room for a fifth. If multiplying national societies will produce a united, harmonious profession, there is great hope for us. But until this be demonstrated we shall stick to the old ship, even though she may leak a little. Before we take to the raft we will stand a while at the pumps.

Dr. Frank Abbott authorizes the statement that his name was signed to the call for this meeting without his knowledge or consent.—Editor.

### NEW JERSEY STATE DENTAL SOCIETY.

The fourteenth annual session of the New Jersey State Dental Society will be held at Asbury Park, commencing at ten o'clock Wednesday morning, July 16th, and continue in session three days. Several papers of great interest to the profession, by members and eminent practitioners of sister societies, will be read. Many promises have been received from dental dealers of the exhibition of new appliances, and every inducement will be given inventors in furthering the exhibition of anything new or useful to the profession by consulting Dr. J. W. Scarborough, Lambertville. The headquarters will be at the Coleman House, the largest and most commodious in the Park, directly fronting and within fifty feet of the surf, having a large hall for the meeting, open on all sides, connected with the hotel. A delightful situation, cool, easy of access from all parts of the State —Philadelphia and New York — low rates and excellent cuisine. The profession generally, members or not, are most cordially invited to meet with us, and spend three days of recreation and profit.

CHAS. A. MEEKER, D. D. S., Sec'y.

NEWARK, N. J., June 9, 1884.

---

### PENNSYLVANIA STATE DENTAL SOCIETY.

The sixteenth annual session of the Pennsylvania State Dental Society will be held at Wilkesbarre, Pa., July 29th, 30th, and 31st, 1884.

Wilkesbarre is situated on the north branch of the Susquehanna River, in the beautiful and picturesque valley of Wyoming.

Hotel accommodations are equal to any in the State, the Wyoming Valley hotel having accommodations for three hundred guests. Rates at this house have been reduced from $3.50 to $2.50 per day, to delegates and their families. Excursion tickets can be had over the following roads: The Lehigh Valley R. R. will sell tickets to Wilkesbarre July 21st and 22d, good to the 26th inclusive, without special order. The Bloomburg Division of the Delaware, Lackawanna and Western will sell regular excursion tickets from all stations on the line. The Philadelphia and Reading, the Pennsylvania and branches, will sell special tickets over their lines. Orders for which (or other information) can be had by addressing

W. H. FUNDENBURG, Cor. Sec'y,
958 Penn Ave., Pittsburg, Pa.

---

### DENTISTS' BENEVOLENT ASSOCIATION.

The first annual meeting of the DENTISTS' BENEVOLENT ASSOCIATION will be held at Sweet Springs, Salina Co., Mo., Thursday, July 10, 1884.

This Association is composed exclusively of dentists and dealers in dental materials, and has for its object financial aid and relief to the families of deceased members. Every member of the profession should be interested in such a movement, and is cordially invited to attend and lend a helping hand toward future success.

As the meeting takes place during the session of the Missouri State Dental Society, arrangements have been made for reduced railroad and hotel rates. Any one desiring to attend is requested to communicate with the Secretary.

C. H. DARBY, President,

St. Joseph, Mo.

R. I. PEARSON, Secretary,

2206 Troost Ave.,

Kansas City, Mo.

---

### NATIONAL ASSOCIATION OF DENTAL EXAMINERS.

The second annual meeting of the National Association of Dental Examiners will be held at Saratoga Springs, N. Y., on Friday evening, Aug. 8, 1884.

All Boards of Examiners are urgently requested to send representatives to this meeting, as matters of great importance are expected to be brought before it.

GEO. H. CUSHING, Secretary.

### SOCIETY MEETINGS FOR JULY, 1884.

Wisconsin State—Third Tuesday, at Milwaukee.
New Jersey State—Third Wednesday, at Asbury Park.
Pennsylvania State—Last Tuesday, at Wilkesbarre.
First District New York—New York, first Tuesday evening.
Missouri State—Sweet Springs, second Thursday.
Chicago City—First Tuesday evening.
Northwestern—Grand Forks, Dak., third Tuesday.
Odontological—New York, third Tuesday evening.
Odontographic—Philadelphia, first Wednesday evening.
St. Louis City—First Tuesday evening.
Washington City—Second Tuesday evening.
American Dental Association—Saratoga, August 5th.

---

### CHANGES PRODUCED IN THE TEETH BY SYPHILIS.

(*From Abstract in Jahrb. f. Kinderh.*, B. XX. H. 4.)

Among the phenomena which are noticeable in connection with hereditary syphilis, are certain ones which are referable to the formation of the teeth. 1. They may be late in making their appearance. Demarquay records a case in which a child, four and one-half years of age, had not had any. 2. Certain changes in their structure are observable. Among these may be mentioned erosions, as the so-called nail marks of Hutchinson. These erosions are crescent shaped, and are located upon the border of the upper central incisors; also microdontism, or dwarfed condition of the teeth; amorphism, in which there are peculiarities in each group—susceptibility to injury which causes them quickly to wear out or to fall out.—*Archives of Pediatrics.*

---

### AMERICAN DENTAL ASSOCIATION.

The twenty-fourth annual session of the Americal Dental Association will be held at Saratoga Springs, N. Y., commencing at 10 A. M., on Tuesday, Aug 5, 1884.

GEO. H. CUSHING, Rec. Secretary.

(11.) I am glad you have a question department, for I believe it a very profitable part of your already excellent journal. Much useful information may come from it that many of your readers desire. I would like to obtain a formula for making good gold solder that will flow easily, and not eat into the plate. Will somebody please furnish such a formula? F. L.

---

(12.) Is it, as a rule, of any benefit to a child to fill the deciduous teeth after the pulps are dead? Are they not more likely to give trouble if filled, than if the cavities are left open? D. D. S.

---

(13.) I desire to ask whether, from a professional standpoint, tobacco is regarded as beneficial to the teeth? If it be, do the advantages outweigh the manifold disadvantages? I am not a practicing dentist, but a woman who reads your journal quite regularly. I am debarred by my sex from obtaining any possible benefits from tobacco. I have frequently heard it asserted that it preserves the teeth, and would be glad to know what is the prevailing professional opinion upon this subject. M. W. L.

---

*Answers.*—In reply to question No. 7, in the June number of the INDEPENDENT PRACTITIONER, let me refer to the fact that fillings of gold and tin, introduced by our old first-class operators, have stood side by side for years, harmoniously tolerating each other's presence, and with no apparent evidence of mischief from chemical or galvanic conflicts. I have also used Robinson's tin mats for lining the cervical walls of such cavities, and then completing with gold foil. In such cases I feel sure of a more perfect adaptation of the filling to the calcareous walls, and safer and more durable stoppings than when gold is used.

C. E. F.

---

In answer to "L." (No. 9), in the last number of the INDEPENDENT PRACTITIONER, I would say that "Electric" gold was so named because it was primarily designed to be used with the electric mallet, although it has since been found desirable for either hand or hand-mallet pressure.

Its peculiarities are cohesiveness in the highest degree, combined with softness under the instruments, together with a very convenient form for using.

ONE WHO USES IT.

---

Inquirer (No. 10) desires information concerning the administration of anæsthetics *per rectum*. This has been engaging the attention of medical men lately, but it is not meeting with unqualified commendation. The method is to introduce a tube connected with the ether or chloroform bottle some distance into the rectum. The bottle is then placed in water heated to about the boiling point of the anæsthetic. The vapor passing into the large intestine is absorbed, and in a few moments the odor may be detected in the breath. It is claimed that anæsthesia may be obtained without any of the bronchial irritation or nausea induced by the usual administration, and there is no "excitement stage." But such severe intestinal irritation has in a number of cases supervened, that the method is not gaining ground. ED.

